# All-optical phase modulation in a cavity-polariton Mach–Zehnder interferometer

**DOI:** 10.1038/ncomms4278

**Published:** 2014-02-11

**Authors:** C. Sturm, D. Tanese, H.S. Nguyen, H. Flayac, E. Galopin, A. Lemaître, I. Sagnes, D. Solnyshkov, A. Amo, G. Malpuech, J. Bloch

**Affiliations:** 1 Laboratoire de Photonique et de Nanostructures, LPN/CNRS, Route de Nozay, 91460 Marcoussis, France; 2 Universität Leipzig, Institut für Experimentelle Physik II, Linnéstr. 5, 04103 Leipzig, Germany; 3 Institut Pascal, PHOTON-N2, Clermont Université, Université Blaise Pascal, CNRS, 24 avenue des Landais, 63177 Aubière Cedex, France; 4 These authors contributed equally to this work

## Abstract

Quantum fluids based on light is a highly developing research field, since they provide a nonlinear platform for developing optical functionalities and quantum simulators. An important issue in this context is the ability to coherently control the properties of the fluid. Here we propose an all-optical approach for controlling the phase of a flow of cavity-polaritons, making use of their strong interactions with localized excitons. Here we illustrate the potential of this method by implementing a compact exciton–polariton interferometer, which output intensity and polarization can be optically controlled. This interferometer is cascadable with already reported polariton devices and is promising for future polaritonic quantum optic experiments. Complex phase patterns could be also engineered using this optical method, providing a key tool to build photonic artificial gauge fields.

## Abstract (web summary)

Quantum fluids such as cavity-polaritons show nonlinear optical properties of interest in applications such as quantum optics. Here, Sturm and colleagues demonstrate an optical control of the phase of a polariton flow, and make use of this to realize a compact exciton–polariton interferometer.

## Graphical abstract

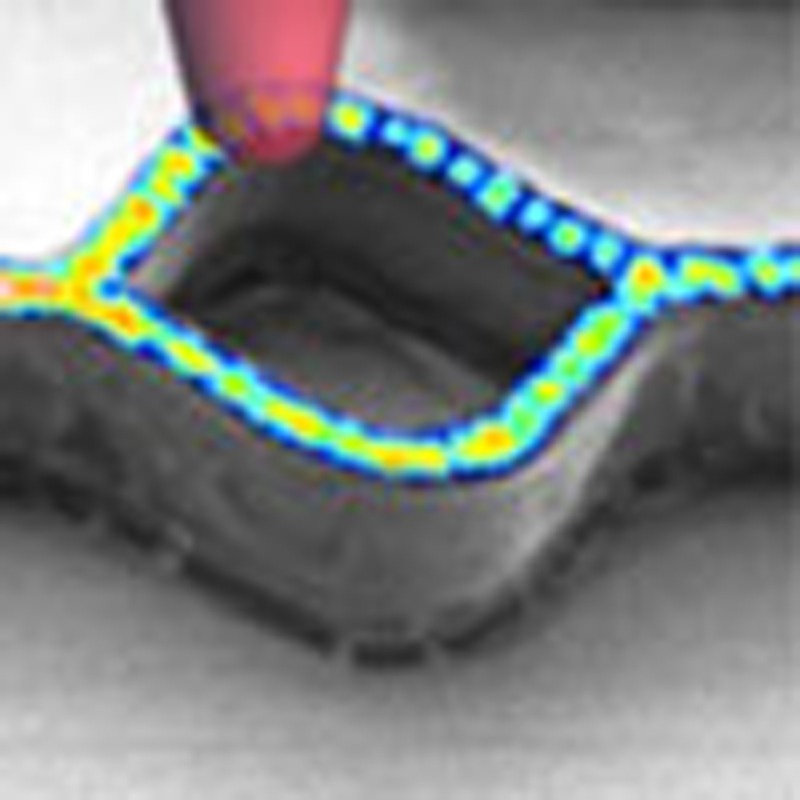

Optical manipulation of quantum fluids has provided extraordinary opportunities to test fundamental properties of matter and to create solid-state simulators^1^. Since the first observations of Bose–Einstein condensation of cold atoms^2^,^3^, optical fields have been successfully used to create controlled potential barriers allowing the study of superfluidity^4^,^5^, the superfluid–Mott transition^6^ or the Anderson localization of matter waves^7^,^8^. In photonic systems, the combination of *χ*^3^ nonlinearities with strong fields has allowed the engineering of optical lattices for photons which behave as photonic simulators of, for instance, graphene physics^9^.

While in these experiments optical fields were used to shape the potential landscape of quantum fluids, they can also be used to locally manipulate the phase of the fluid. One of the most remarkable examples is the use of Raman transitions in which atoms gain a controlled phase when going from site to site in lattices, which has allowed the engineering of gauge fields^10^,^11^ and opens new avenues to test Quantum Hall physics with neutral particles^12^.

An emerging class of interacting spinor quantum fluids that would strongly benefit from all-optical phase manipulation is that of cavity polaritons^13^. They are light-matter quasi-particles arising from the strong coupling regime between the optical mode of a Fabry–Perot cavity and excitons confined in quantum wells^14^. The photonic part enables ballistic polariton propagation over macroscopic distances with a speed of the order of a few percent of the speed of light^15^, whereas their matter part is responsible for strong polariton interactions with their environment^16^,^17^,^18^,^19^. Fascinating fundamental properties of cavity polaritons have been revealed by optical spectroscopy^20^, such as superfluidity^21^,^22^ or the formation of topological defects like quantized vortices^23^,^24^, solitons^25^,^26^ or magnetic monopoles^27^. Because of their giant nonlinearity, polaritons are also very promising for the implementation of innovative opto-electronic devices^28^.

The first demonstration of the potentiality of cavity-polaritons for opto-electronic devices came with the realization of polaritonic parametric oscillator^29^ and amplifier^30^, where the *χ*^3^ nonlinearity was found to be orders of magnitude larger than in any other optical systems. Low threshold bistability^31^ and multistability^32^,^33^ have been demonstrated together with several schemes of optical switches^34^,^35^, optical gates^36^,^37^, polariton transistors^36^,^38^ and resonant tunnelling diode^39^. This rapid recent development together with flourishing theoretical proposals for more complex architectures reveal the potential of cavity polaritons as a new platform for integrated photonics, where coherent emission, optical guiding and nonlinearity can be combined within the same chip.

An interesting possibility of manipulating a polariton flow is to use an external potential created by a non-resonant optical pumping^17^,^40^,^41^. The pump locally creates carriers or excitons which interact directly with polaritons, inducing a potential barrier *V* able to reach several meVs. This optically induced potential has been used so far to confine polaritons in well-defined regions of space^40^,^41^, to block a polariton flow^38^, to create an artificial defect^42^ or to blueshift a quantized polariton quantum state^39^.

Here we make use of this optically induced potential in a new way and control coherently the phase of a polariton flow. The quantum fluid propagates with a kinetic energy *E*_*k*_ larger than *V,* so that it is transmitted across the barrier but slowed down in the barrier region. As a result the polariton flow acquires a phase shift *δφ* as compared with the case without barrier.

Considering the simplest case of a square shape potential barrier, the induced phase shift difference is given by:


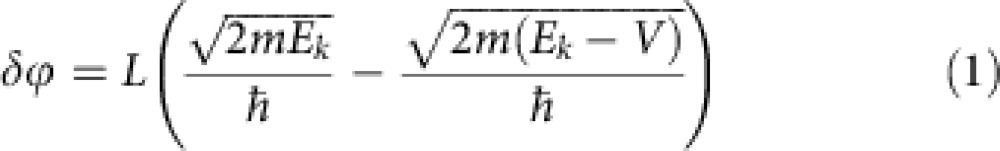


where *m* is the polariton effective mass and *L* the width of the optically induced potential. For realistic parameters, such as *E*_*k*_=1.5 meV, *V*=1 meV and a polariton mass of 4 × 10^−5^*m*_0_, a *π* phase shift can be obtained within 6 μm only. This large value can be reached because the polariton kinetic energy is comparable with the height of the potential barrier. In other words, thanks to the quantization of the cavity mode, we are dealing with highly dispersed ‘slow light’^43^ for which the impact of a weak barrier, amplified by strong coupling, is maximal.

Here, we demonstrate experimentally this pronounced phase shift and use it to implement all-optical polariton interferometers. By slowing down the polariton flow in one of the arms of a Mach–Zehnder interferometer (MZI), we can control the intensity of the output flow and its polarization. These original functionalities arise from the mixed light-matter nature of cavity polaritons and are implemented in an integrated micron-sized circuit.

## Results

### Two types of polariton interferometers

**Figure 1:**
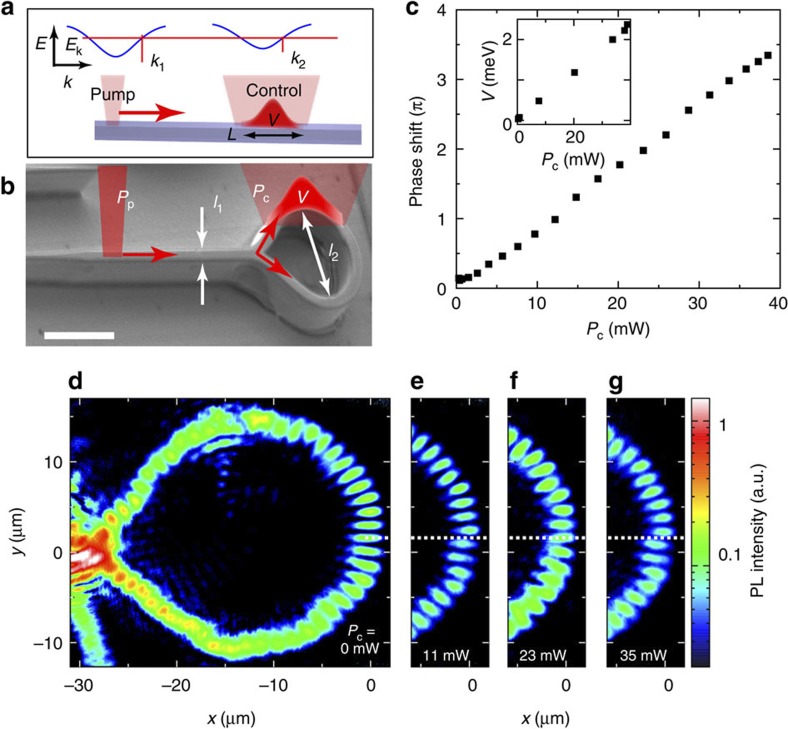
Sagnac interferometer. (**a**) Schematic of the mechanism used to slow down the polariton flow. (**b**) SEM image of the SI; the white scale bar corresponds to a length of 20 μm and *l*_1_ and *l*_2_ corresponds to 3 and 25 μm, respectively. (**c**) Measured phase shift as a function of *P*_c_. The uncertainty is about ±5 × 10^−2^ *π*; inset: measured potential height *V* as a function of *P*_c_. (**d**–**g**) Real-space imaging of polariton emission in the SI device for *P*_c_=0 mW, 11 mW, 23 mW and 35 mW.

Two types of polariton circuits, namely a Sagnac interferometer (SI) and a MZI have been fabricated, using electron beam lithography and dry etching of a high-quality GaAs/AlGaAs microcavity (see Methods). Figure 1b shows a scanning electron microscopy (SEM) image of the microcavity sculptured in the shape of a SI.

### Optically controlled phase shift in a SI

We first discuss the operating regime of the SI, illustrating the ability to optically change the phase of a polariton flow. The input of the SI is excited by a non-resonant *cw* laser focused on a 2 μm diameter area with a pumping power above the threshold for polariton condensation. This pumping scheme has been shown previously to generate a mono-kinetic flow of coherent polaritons (with kinetic energy typically of the order of 2.5 meV)^40^. This flow splits in two propagating beams when entering the SI, which interfere in the opposite part of the interferometer. The coherence of the condensate is preserved during the propagation and the resulting interference fringes can be directly observed when spatially imaging the polariton emission as shown in Fig. 1d. To change the phase of the polariton flow in the upper part of the SI, a control laser beam is focused on the upper part with a spot size around 7 μm. This laser is strongly blue detuned with respect to the polariton resonances (non-resonant excitation) and locally injects a cloud of uncondensed excitons. Because of repulsive polariton–exciton interactions, this reservoir induces a local blueshift of the polariton energy states, thus creating a potential barrier with a spatial profile determined by the shape of the control beam and a height proportional to the exciton density (Fig. 1a)^17^,^40^. Probing locally the polariton luminescence in the region of the control beam, we can measure the induced local blueshift and thus deduce the height of the barrier for different values of the control beam power *P*_c_ (inset in Fig. 1c). In our experiments, this potential barrier is kept smaller than the kinetic energy of the incident flow. Thus polaritons are not stopped by this potential barrier but can pass across. In this region, the polariton flow is slowed down (see Supplementary Note 1 and Supplementary Fig. 1) and thus acquires a phase shift as compared with the case without this barrier. Fig. 1d–g present the interference patterns obtained for increasing power *P*_c_ of the control laser beam. The induced phase shift is directly highlighted by a progressive displacement of the interference fringes and reaches values as large as 3*π* (see Fig. 1c).

### Transmission–modulation in a MZI

**Figure 2:**
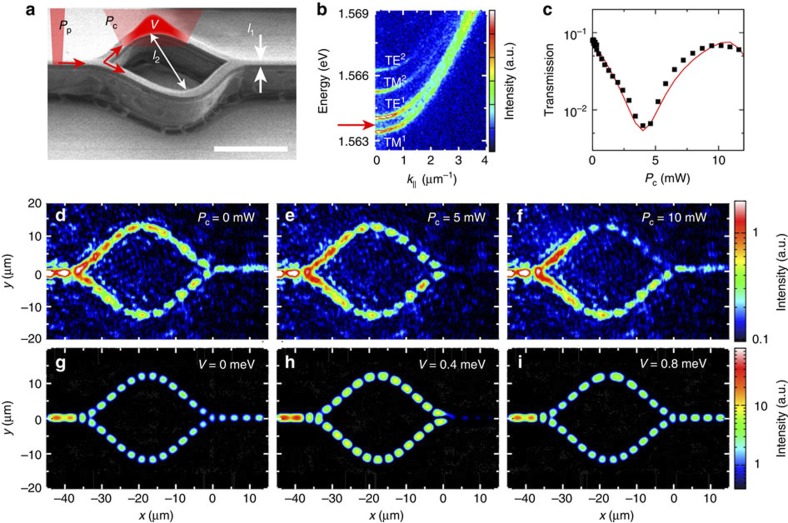
Modulation of the MZI transmission. (**a**) SEM image of the polariton MZI; the white scale bar corresponds to a length of 20 μm and *l*_1_ and *l*_2_ corresponds to 3 and 25 μm, respectively. (**b**) Measured polariton dispersion. The red arrow indicates energy of injected polariton flow. (**c**) (black squares) Measured transmission as a function of *P*_c_ (red line) calculated transmission using equation (1) assuming a rectangular potential profile and a polariton lifetime of 20 ps. (**d**–**f**) Spatially resolved polariton emission measured for different values of *P*_c_; (Note that the modulation of the signal at the output is probably caused by disorder) (**g**–**i**) Calculated emission pattern for different heights of a Gaussian (*σ*=4 μm) induced potential (parameters: *E*_*k*_=0.8 meV, *m*=4 × 10^−5^ *m*_0_). (The fringes in the output are caused by polaritons backscattered from the end of the device.)

Since a large phase shift can be induced with a control region of only a few microns, this mechanism can be used to control optically the output polariton beam of a small-sized polariton MZI (see Fig. 2a). As we will explain in the following, depending on the precise energy of the incident polariton flow, interesting polarization effects can be demonstrated. The underlying physics arises from the complex band structure of one-dimensional microcavities. Because of the strain induced by etching of such anisotropic microstructures, the polariton lateral confinement gives rise to the formation of several 1D polariton sub-bands which are linearly polarized (Fig. 2b). The lowest energy sub-band is linearly polarized along the wire (usually named TM polarization) while the second one is linearly polarized perpendicular to the wire (and usually named TE polarization)^44^.

Thus to properly investigate the operating regimes of the MZI, we want to precisely control both the kinetic energy and the polarization of the incident polariton flow. For this purpose, we inject a monochromatic polariton flow in the input arm of the MZI using a resonant monomode laser beam. The energy of the polariton flow is that of the exciting laser, while the polariton in-plane wavector gets fixed according to the polariton dispersion.

We first consider resonant injection of TM polaritons with energy below the bottom of TE^1^ sub-band (*cf*. Fig. 2b). The effect of the control laser beam is illustrated in the real-space images of the polariton emission shown in Fig. 2d–f and in Supplementary Movie 1. For *P*_c_ = 0, the signals propagating in the two arms interfere constructively at the device output, allowing maximum transmission of the device (Fig. 2d). Switching on the control laser, strong modulation of the output intensity is observed. To quantitatively evaluate the MZI transmission, we measure the integrated intensity *I*_out_ (respectively, *I*_in_) in a portion of length 5 μm located in the output (respectively, input) arm. The extracted transmission *T* = *I*_out_/*I*_in_ is shown in Fig. 2c as a function of *P*_c_. A pronounced transmission decrease by one order of magnitude is achieved for *P*_c_ = 5 mW, corresponding to an induced phase shift of *π* and signature of destructive interference between polaritons flowing in each arm. The extinction ratio is limited by the background scattered laser light (speckle pattern visible on Fig. 2d–f). This ratio could be significantly improved when operating the device in transmission geometry (see Supplementary Note 2 and Supplementary Fig. 2). Maximum transmission is recovered for *P*_c_ =10 mW, corresponding to a 2*π* phase shift induced by the control beam. Note that in all the measured real-space images, pronounced fringes are observed both at the input and in the arms of the MZI. They are caused by interferences between incident polaritons and polaritons backscattered at the input and output of the ring. These reflections together with the losses introduced by the finite polariton lifetime reduce the maximum transmission achieved with the device. The behaviour of our interferometer is reproduced using a simulation based on a 2D spinor Schrödinger equation (Fig. 2g–i). We consider the propagation of a coherent wave in a guiding potential, which defines the shape of the circuit, and we introduce a potential barrier of varying height to mimic the effect of the control laser beam. The simulations fully reproduce all the interference patterns experimentally observed. This experiment demonstrates that, taking advantage of the strong exciton–polariton interaction, we can drive the polariton interferometer with an optical beam acting on a region as narrow as 7 μm.

### Polarization switching in a MZI

**Figure 3:**
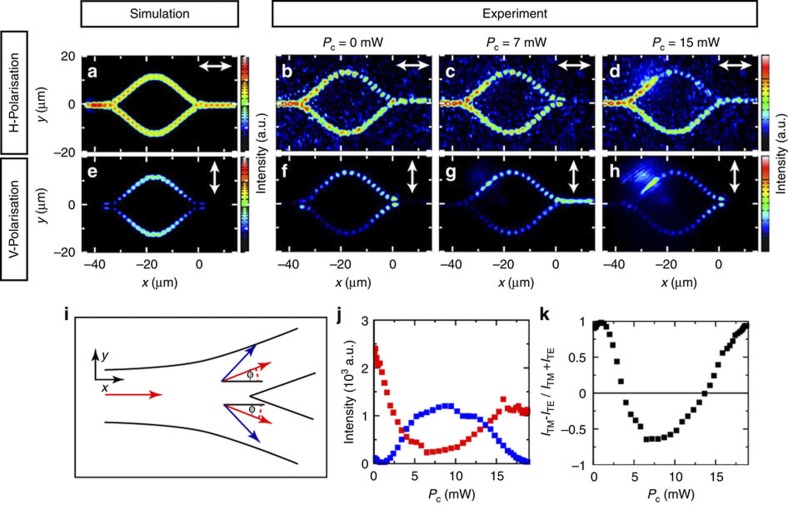
Polarisation conversion. (**a**,**e**) Calculated pattern for the emission of horizontally (**a**) and vertically (**e**) polarized polaritons for a splitting between the TE^1^–TM^1^ bands of 
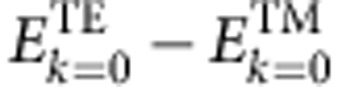
=0.4 meV, *E*_*k*_=1.85 meV, *m*=4 × 10^−5^*m*_0_ without any induced potential. The white arrow indicates the direction of the investigated polarization in each case; (**b**–**d**,**f**–**h**) Spatially resolved polariton emission pattern for the horizontal (**b**–**d**) and vertical (**f**–**h**) polarization for different values of *P*_c_ and *E*_*k*_=1.2 meV, thus corresponding to an incident polariton energy between the minima of the TE^1^ and TM^2^ sub-bands. (**i**) Scheme of the effective magnetic field at the junction. The direction of the wave vectors and the effective magnetic field is represented by red and blue arrows, respectively. (**j**) Measured transmitted intensity in (red) TM and (blue) TE polarization as a function of *P*_c_. (**k**) Degree of linear polarization of the output beam as a function of *P*_c_.

We now want to show that the splitting between TE- and TM-polarized sub-bands is responsible for a precession of the polariton polarization within the MZI, and provides a tool to control the polarization of the output beam. To evidence this effect, we inject TM-polarized polaritons with energy above the bottom of the TE^1^ sub-band. Figure 3b (respectively, 3f) shows the emission intensity measured along the device for horizontal (respectively, vertical) polarization when *P*_c_=0. For horizontal polarization, we observe the same feature as what was described just before: the TM (horizontally)-polarized incident polariton flow propagates all along the MZI with constructive interferences at the output arm. Interesting features appear when measuring the emission under vertical polarization. Since the polarization of the incident polariton flow is horizontal, no emission is found in the input arm in vertical polarization. Nevertheless it appears clearly that vertically polarized polaritons are injected within the two MZI arms. The shape of the fringe pattern at the input of the ring, with a two transverse lobe structure indicates a *π* phase shift between polariton flowing in the upper and lower arms, for vertical polarization. As a result, destructive interference occurs at the output of the ring and no output signal is measured under vertical polarization.

This phenomenon can be understood as follows. The splitting between the TE and TM sub-bands can be described as an effective magnetic field 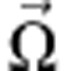 acting on the polariton pseudo-spin 
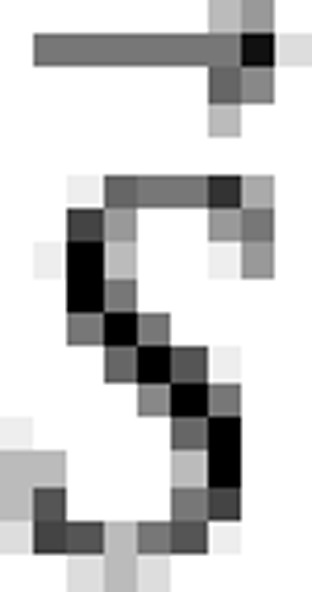
 (ref. 45). The orientation of the field is given by (cos 2*φ*, sin 2*φ*), where *φ* is the angle that the wire makes with the horizontal axis (*x* axis). Polaritons injected with a horizontal (TM) polarization in the input of the device have their pseudo-spin aligned along the effective magnetic field, and thus propagate preserving their polarization. At the junction point, they split into two flows with opposite values of *φ* (positive (respectively, negative) for the upper (respectively, lower) arm). Since horizontal polarization is no more aligned along the effective magnetic field in the upper and lower arms, the polariton pseudo-spin starts precessing according to the equation: 
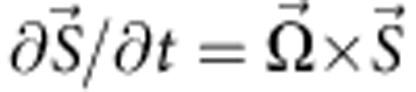
. As the vertical projection of the effective magnetic field has opposite signs in the two arms, the rotation directions of the pseudo-spin are opposite. In one arm, the precession results in the appearance of right-circular polarization, whereas in the other one left-circular polarization is created. Thus, there is a *π* phase shift between the vertically polarized polariton flows in the upper and lower arms, resulting in destructive interferences at the output of the device (see Fig. 3f). Simulations using the model described above reproduce well this polarization feature (see Fig. 3a,e).

We can use this precession effect to control the polarization of the output of the interferometer, as illustrated in Fig. 3 and in Supplementary Movie 2. When a *π* phase shift is induced between the upper and lower MZI arms, destructive interferences occur for horizontal polarization (for *P*_c_=7 mW as seen in Fig. 3c), whereas for vertical polarization the total phase between the two arms becomes *π*+*π*=2*π* and constructive interference occurs in the output arm (see Fig. 3g). Thus switching of the polarization is obtained in the output signal, and vertically, that is, TE, polarized polaritons come out from the device. At higher power of the control laser, when a 2*π* phase shift is induced for the horizontal polarization, the output signal recovers TM polarization. Figure 3k summarizes how the linear polarization ratio changes sign with increasing *P*_c_. Note that a polariton switch^34^ has already been reported using the polariton bistability^32^,^33^ and their spin-dependent interaction^46^,^47^. One advantage of the method we propose here is that the control beam relies on a non-resonant excitation, and is thus less sensitive to fine energy tuning.

## Discussion

We can also demonstrate switching between transverse modes when pumping the system above the third polariton band (see Supplementary Note 3 and Supplementary Fig. 3). More complex schemes, based on the present mechanism of phase modulation, can be easily envisaged. We simulate a router based on a polariton MZI with two output leads (see Supplementary Note 4 and Supplementary Fig. 4). Regarding the switching time, we expect it to be comparable with the lifetime of excitons within the reservoir. Previous reports on time-resolved measurement on cavity polaritons give an estimate of the reservoir lifetime in the 100 ps range^48^.

In conclusion we have realized polariton devices based on polariton circuits. Operating in the linear regime, the device allows guiding a coherent polariton flow into a ring, splitting this flow into two and manipulating the phase of one of them using a local optical excitation. We demonstrate strong phase and polarization modulation, based on the simultaneous achievement of slow light effect and strong exciton–photon coupling. This device provides a building block for a new platform for all-optical signal processing, and can be put in cascade with other recently developed polariton devices^39^. This first proof of principle opens the way towards more complex polariton devices, for example, optical gates based on interferometry^49^.

Our polariton device can be also very interesting for more fundamental researches. For instance, when applying an external magnetic field, a polariton Berry phase arises from the interplay between the TE–TM splitting and the Zeeman splitting. Polaritons travelling in the interferometer clock and anticlockwise would acquire a different Berry phase and thus interfere differently when scanning the magnetic field^50^. Our interferometer is also very promising for quantum optics experiments like the generation of non-classical states^51^. Recently we reported a polariton device based on nonlinear resonant tunnelling across a small 0D island^39^. This device could be optimized in the near future to operate in the quantum regime. Because of single polariton nonlinearity and polariton blockade^52^, polaritons would cross the island one by one thus generating a non-classical polariton flow in the output of the device. The present polariton interferometer could be put in cascade with this resonant tunnelling diode and would thus provide a quantum platform to realize single polariton interferometry, and which-path experiments^53^. For such challenging experiments, reduction of the losses is certainly an important issue. Several methods can be foreseen, namely further improvement of the cavity Q factor by increasing the number of pairs in the mirrors, reducing the size of the interferometer, or even switching to polaritons propagating beyond the light cone^54^.

An interesting perspective is also to use the strongly spin-dependent polariton interactions to induce spin-dependent phase shifts. Indeed, if the excitonic reservoir is spin polarized by resonant excitation with circularly polarized light, the induced potential barrier will be mainly felt by polaritons of the same spin as the reservoir. This can be used to induce spin-dependent phase shifts in a wire, and also to engineer spin-dependent phase shifts in the tunnelling of polaritons in a lattice. Spin-dependent tunnelling has been recently proposed as a route to engineer spin–orbit coupling^55^ and to fabricate artificial gauge fields for photons^56^,^57^ and we provide a realistically feasible mechanism to realize it.

## Methods

### Sample design and experimental set-up

The sample consists of a *λ*/2 Ga_0.05_Al_0.95_As cavity embedded between two Ga_0.05_Al_0.95_As/Ga_0.8_Al_0.2_As Bragg mirrors with, respectively, 40 (28) pairs in the bottom (top) mirror. The nominal quality factor is around 10^5^. Three sets of four 7 nm GaAs quantum wells are inserted at the antinodes of the electromagnetic field resulting in a 15 meV Rabi splitting. The SI and MZI were designed using electron-beam lithography and dry etching. In both structures, the diameter of the ring was chosen to be 25 μm and the width of the wires as well as of the ring is 3 μm. The exciton–photon detuning defined as *E*_*C*_ (*k*=0)−*E*_*X*_ (*k*=0) is equal to −4.5 meV (respectively, −9 meV) for the SI (respectively, MZI) structure.

Micro-photoluminescence measurements are performed at 10 K. The excitation is provided by two single mode Ti:Sapphire lasers. The far (respectively near) field spectroscopy is obtained projecting the Fourier plane of the detection microscope objective (respectively, the sample surface plane) on the input slit of a monochromator, placed prior to a nitrogen-cooled CCD camera.

### Simulation using 2D spinor Schrödinger equation

We simulate a polariton flow inside a 2D waveguide structure corresponding to the experimental interferometer structure. Along one arm of the interferometer, we consider an induced 2D Gaussian-shaped potential with *σ* =4 μm. The height *V* of the potential barrier acting on polaritons is taken *V*=|*χ*|^2^*n*_*x*_6
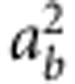
*E*_b_, where |*χ*|^2^ is the exciton fraction of the polariton, *n*_*x*_ is the exciton density in the reservoir per quantum well, *a*_b_ is the exciton Bohr radius and *E*_b_ the exciton binding energy.

The splitting between transversal and longitudinal polaritonic modes is reproduced in the model by the introduction of an effective in-plane magnetic field. It acts on the polariton pseudo-spin, generating a Zeeman splitting between polaritonic states of different polarization and a sub-band structure analogous to the experimental one.

## Author contributions

C.S., D.T, H.S.N., A.A. and J.B. designed and performed the experiment as well as analysed the data. H.F., D.S. and G.M. carried out the simulations. E.G., A.L. and I.S. grew and etched the sample. All of the authors discussed the underlying physics and contributed to the manuscript.

## Additional information

**Accession numbers:** Sequence data have been deposited in the GenBank/European Molecular Biology Laboratory databases under the following accession codes: AT1G31770 (*AtABCG14*), AT3G18780 (*ACTIN 2*), At4G05320 (*UBIQUITIN 10*), AT3G48100 (*ARR5*), AT4G37490 (*CYCB1;1*).

**How to cite this article:** Sturm, C. *et al.* All-optical phase modulation in a cavity-polariton Mach–Zehnder interferometer. *Nat. Commun.* 5:3278 doi: 10.1038/ncomms4278 (2014).

## Supplementary Material

Supplementary Information Supplementary Figures 1-4 and Supplementary Notes 1-4

Supplementary Movie 1 Transmission of the MZI. (top) Measured transmission as a function of Pc. (bottom) Real space images of the polariton emission modulation for increasing values of Pc. The excitation conditions are schematically shown in Figure 2b.

Supplementary Movie 2 Polarization Switching.(top) Linear degree of polarization measured on the MZI output as a function of Pc. IH and IV correspond to the horizontal and vertical linear polarization. (bottom) Spatially resolved degree of linear polarization measured on the MZI for increasing values of Pc. The excitation conditions are schematically shown in Figure 3a.
