# Supplementary material for: All-optical phase modulation in a cavity-polariton Mach–Zehnder interferometer
Source: Nat Commun. 2014 Feb 11;5:3278. doi: 10.1038/ncomms4278 (PMC3926000; doi:10.1038/ncomms4278)
Supplement: Supplementary Information — Supplementary Figures 1-4 and Supplementary Notes 1-4 [file ncomms4278-s1.pdf]

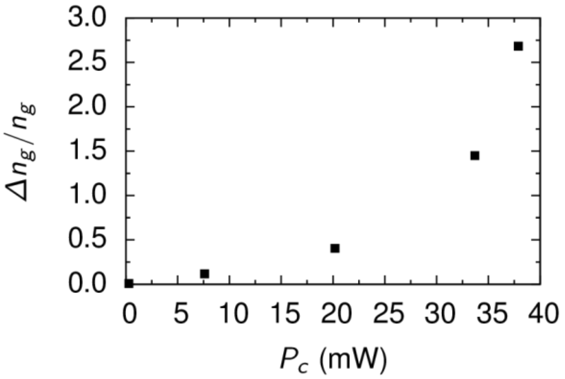

Supplementary Figure 1: Group Index. Change of the group index induced by the control potential.

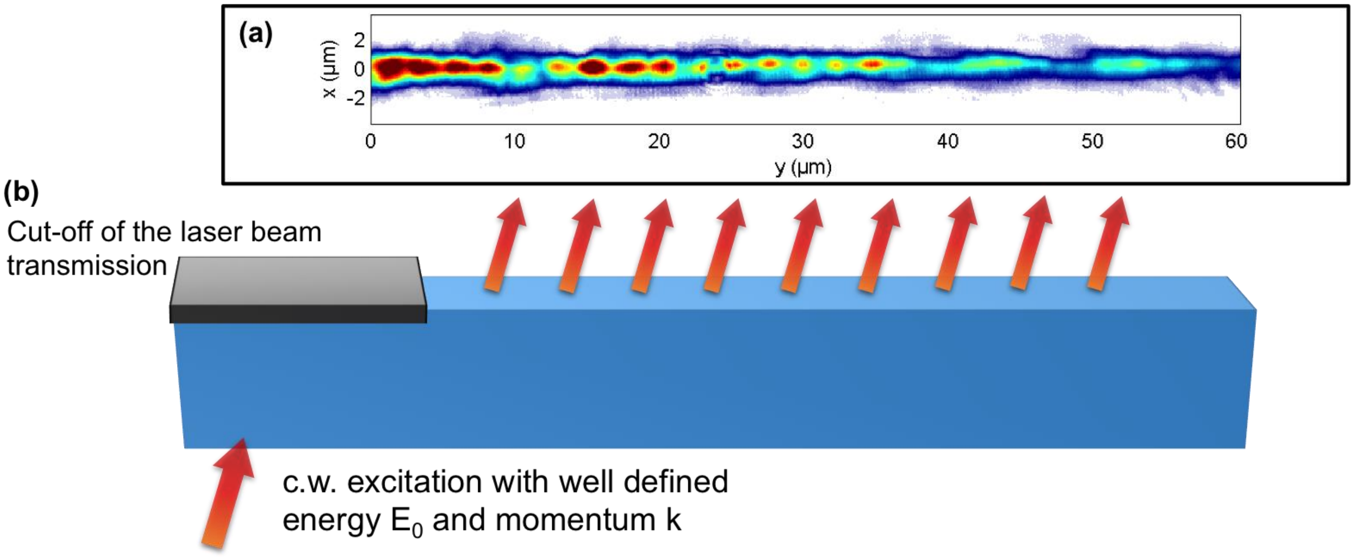

Supplementary Figure 2: Cavity-polaritons in transmission geometry (a) Real space imaging of the polariton emission from a wire in the transmission geometry at T = 10K. **(b)** Schematic of the experiment.

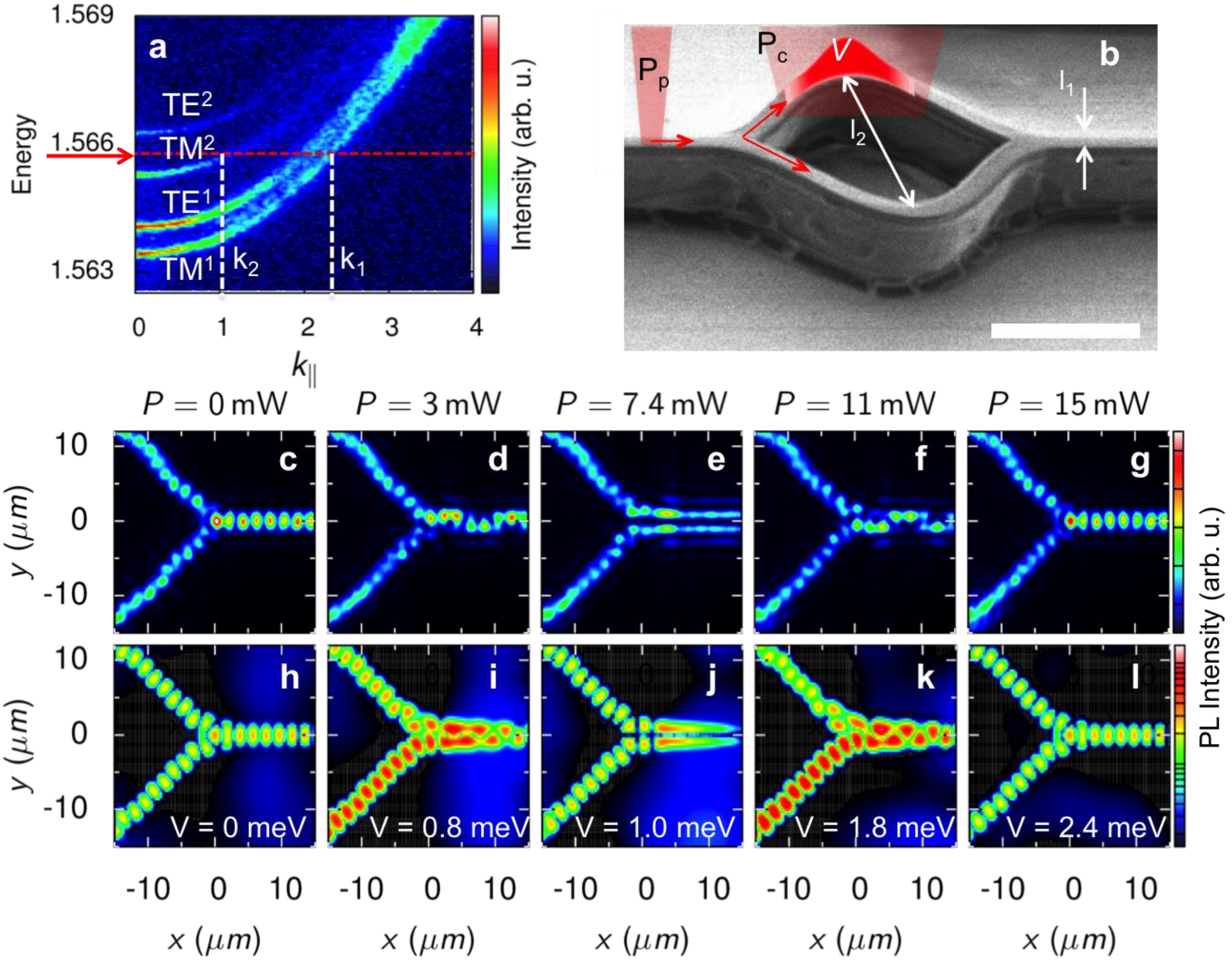

Supplementary Figure 3: Switch between transverse modes (a) Measured polariton dispersion showing with red arrow the energy of injected polariton in the present experiment. The white dashed lines indicates the two main wave vectors (k_1 and k_2) of the first and second order 2D confined polariton modes **(b)** Schematic of the performed experiment; the white scale bar corresponds to a length of 20μm and l_1 and l_2 corresponds to 3 μm and 25μm, respectively. **(c-g)** Spatially resolved emission for five values of P_c; **(h-l)** Calculated emission pattern for different heights of the potential barrier (Parameters: $E_k = 3.2\,\text{meV}$, $m = 4\cdot 10^{-5} m_0$, σ = 4 μm).

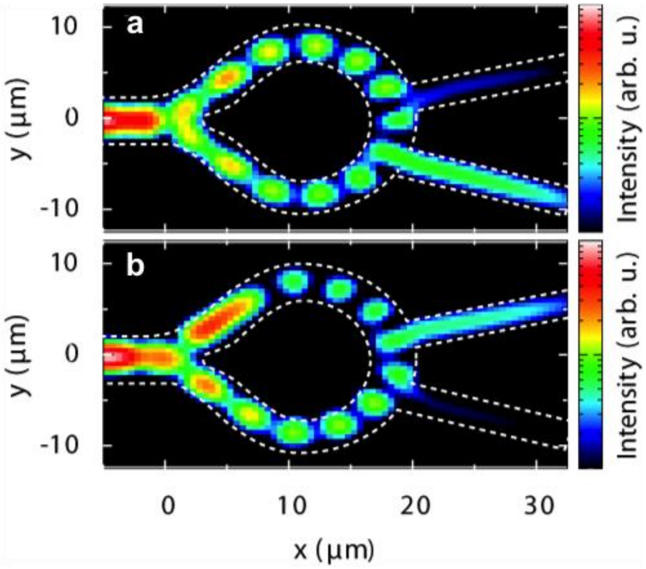

Supplementary Figure 4: Polariton Router (**a and b**) Calculated polariton emission in the polariton router for an induced potential of **(a)** V = 0 meV and **(b)** V = 0.8 meV (Parameters: $E_k = 0.9\,\mathrm{meV}$, $m = 4 \cdot 10^{-5} m_0$, σ = 4 µm). The dashed white lines indicate the design of the proposed router.

Supplementary Note 1: Modulation of the group index

The contribution of the slow light effect to our device can be quantified by define an effective group index for the in-plane propagation as $n_{\mathrm{g}} = c/v_g = \hbar c\,\partial k/\partial E_k$, where c is the speed of light and v_g the polariton group velocity. For a kinetic energy E_k of the order of 2.5 meV (resp. 0.44 meV) one gets n_g = 80 (resp. 190). As explained in the main text, the optically induced potential changes the in-plane k vector and therefore inducing a change in the group index given by:

$$\frac{\Delta n_g}{n_g} = \sqrt{\frac{E_k}{E_k - V}} - 1 .$$

As an example, the experiment shown in Figure 1 corresponds to a change of the group index as large as $\Delta n_g/n_g = 3$ (Supplementary Figure 1). This strong slow light effect induced by the polariton non-linearity explains the giant phase modulation achieved in our devices.

Supplementary Note 2: Observation of 1D cavity-polariton in transmission geometry

The extinction ratio we determined for the Mach-Zehnder interferometer output is limited by scattered laser light. In the main text of the manuscript we propose to improve this by switching to a transmission experiment. Here we show on a wire cavity containing InGaAs quantum wells that this experiment is indeed feasible. In this cavity we resonantly inject polaritons and measure their propagation in transmission geometry (Supplementary Figure 2). It is obvious that, in contrast to the reflection geometry used for the interferometer samples, the detected signal comes only from the propagating polaritons within the wire and the detection of scattered light from the laser beam is suppressed. This suppression of the laser beam is so good that one could even measure directly the transmission within the excitation area.

Supplementary Note 3: Switching between transverse modes:

In Figure 3 we have shown that by injecting polaritons in the MZI above the second polariton subband, we can control the output polarization of our device via the mixing of the lowest TE^1 and TM^1 modes inside the structure. These modes correspond to the lowest transverse modes of each polarization. We are now interested in going a step further and play with the conversion of different transverse modes. Here we inject polaritons in the MZI with energy above the third polariton sub-band (TM^2) (see Supplementary Figure 3a). Such a band corresponds to second order transverse polariton modes with a transverse distribution showing two antinodes with opposite phase, and one node at the center, whereas the lowest subbands (TM^1 and TE^1) are first order transverse modes with an antinode in the center. Because of the gaussian profile of the excitation beam, essentially TM^1 and TE^1 polaritons are injected in the interferometer. These two modes have almost the same k vector k_1, and so they undergo almost the same phase shift along propagation. At the input of the interferometer, part of these polaritons are transferred into the mode TM^2, characterized by much smaller k vector k_2. Because this TM^2 mode presents two out of phase antinodes, it is easy to show that TM^2 polaritons injected in the upper arm are out of phase with respect to TM^2 polaritons injected in the lower arm. As a result, they undergo destructive interference at the MZI output and only first order polariton modes are transmitted when $P_c = 0$ (see Supplementary Figure 3c). When applying the control laser beam, TM^2 starts to be transmitted and spatially oscillating intensity is obtained at the output. When a π phase shift for first order modes is reached, only second order polaritons are transmitted with the characteristic transverse profile (Supplementary Figure 3e). Good transmission of first order polariton modes is recovered for an induced phase shift of 2π on the second order mode (Supplementary Figure 3g). This switching between different transverse modes is reproduced by our simulations as can be seen in Supplementary Figure 3, h–l.

Supplementary Note 4: Proposal for a polariton router:

More complex schemes can be envisaged based on the reported mechanism for polariton phase modulation. Here we propose a polariton router and consider a polariton MZI such as the one described in the paper but with two output leads. Supplementary Figure 4 shows the results of numerical simulations showing that the control laser beam can switch transmission from one output lead to the other. Indeed changing the phase shift in one of the MZI arm moves the position of the interference fringes in the ring. In Supplementary Figure 4a, the upper output lead is aligned with a node of the polariton field whereas the lower one is aligned with an antinode. This explains why transmission essentially occurs from the lower output. Opposite situation is obtained in Supplementary Figure 4b corresponding to a different value of P_c. Thus controlling P_c, on can switch the output signal from one lead to the other.
